# Reliability of teledentistry mobile photos versus conventional clinical examination for dental caries diagnosis on occlusal surfaces in a group of school children: a diagnostic accuracy study

**DOI:** 10.1186/s12903-025-05802-z

**Published:** 2025-04-11

**Authors:** Lobna Sakr, Hala Abbas, Nahwand Thabet, Fatma Abdelgawad

**Affiliations:** 1 Military Medical Academy, Cairo, Egypt; 2 Pediatric Dentistry & Dental Public Health Department, Faculty of Dentistry, Cairo University, Cairo, Egypt; 3 Oral Health Expert FDI, Cairo, Egypt; 4 Pediatric Dentistry and Dental Public Health, Faculty of Dentistry, Cairo University, Cairo, Egypt

**Keywords:** Dental caries, Digitalization, Digital dentistry, School children, Teledentistry

## Abstract

**Background:**

Teledentistry offers an effective, cost-efficient solution for caries detection in school children, improving access to early diagnosis and preventive care in underserved areas. Through remote imaging, it enables timely assessments without in-person visits, helping bridge healthcare gaps and enhance children's oral health outcomes.

**Participants and methods:**

Three examiners participated in this study, examiner 1 (E1) clinically examined the children then examined the clinical photos, examiner 2 (E2) examined the photos only, and examiner 3 (E3) took the clinical photos at school without participating in clinical examination. Sample size consisted of 141 school-going children from primary one to primary six grades. They were examined clinically by E1 using deft/DMFT indices, then clinical photos were taken for both arches from occlusal view. Occlusal surface photos were only included in the analysis. These photos were examined by E1 and E2 after a 48-h wash-out period and the same indices were measured and calibrated.

**Results:**

There was a strong agreement between clinical and photo diagnosis for E1 (kw = 0.899, *p* < 0.001), clinical and photo diagnosis for E2 (kw = 0.834, *p* < 0.001), and image diagnoses (E1) and (E2) (kw = 0.898, *p* < 0.001). The internal consistency between clinical and photo diagnoses (E2) was acceptable 0.790 (0.706:0.85). For other scores, the internal consistency between all variables was excellent (α > 0.9).

**Conclusion:**

Teledentistry provides accurate and reliable results. It is a valuable tool for school-screening programs without dental radiographs.

**Trial registration:**

It was retrospectively registered on clinical trial.gov on 25/10/2024 with an identifier NCT06661837.

## Background

Dental caries is considered an infectious preventable multifactorial childhood disease [1]. When teeth first erupt, they are highly susceptible to dental caries and then they become more resistant to subsequent acid challenges. The clinical significance is that increased attention should be paid to monitoring the caries status of teeth and providing preventive care during dental eruptive stages [2].

Children nutrition and development is strongly affected by the presence of a healthy dentition. Children need their primary teeth in speech, eating and growth, which affects overall quality of life. Therefore, it is essential to prevent caries [3–5]. Children with active caries on their primary teeth during mixed dentition may have distinct microbiological characteristics than children without caries [6].

The restrictions by the COVID-19 pandemic introduced teledentistry to different oral health settings [7]. Teledentistry can serve as an adjunct to direct visual examination and radiographs, as part of a comprehensive evaluation [8]. Therefore it’s considered a screening tool [9]. Awareness and motivation can be raised through sending photos and images of children’s teeth to their legal guardians/parents to seek dental treatment. Teledentistry also eliminates the need for an on-site dentist in screening programs, which saves the workforce and clinical resources. This model can be implemented with only a trained dental assistant [10–12].

Teledentistry has the advantage of providing shift in healthcare paradigm, providing treatment services and, increasing accessibility of dental treatment in rural areas [12, 13].

Panat et al. [14], discovered that teledentistry is an effective strategy for screening school children for dental caries and dividing them according to their caries risk category. This risk assessment can aid dental practitioners in developing therapeutic and preventative programs to battle dental caries.

Kopycka et al. [13] explored the use of intraoral cameras and telehealth technology to screen preschool children for early childhood caries (ECC). Using the Health-e-Access telehealth network, researchers compared dental images captured by intraoral camera with traditional visual oral exams. A calibrated examiner assessed 50 children aged 4 to 6 from an inner-city childcare center, first through direct examination and later via randomized, de-identified images taken by a trained assistant. Results showed that more cases of caries and a higher number of carious teeth were detected through teledentistry (42%) than visual exams (28%), with a mean dfs score of 2.10 versus 1.50.

AlShaya et al. [15] evaluated the effectiveness of teledentistry as a cost-effective solution. Researchers compared the accuracy of caries detection using intraoral photographs taken by a dentist and schoolteachers with clinical examinations of 5–10-year-old children. A calibrated dentist assessed the children clinically and then blindly reviewed the photographs after two weeks. Results showed that mean DMFT scores for primary teeth were similar across clinical, dental-teledentistry, and teacher-teledentistry assessments, with comparable findings for permanent teeth. Sensitivity and specificity were high for both methods, particularly in primary teeth (95% and 94.3% for dentist photographs; 98.3% and 91.4% for teacher photographs).

Teledentistry overcomes physical obstacles that prevent proper healthcare from reaching rural and underdeveloped areas [16]. Teledentistry is widely utilized in rural communities, enabling the transmission of data, images, and audio-visual content to facilitate diagnosis and consultations between individuals separated by distance [17].

Intraoral photographs offer several advantages over traditional clinical examinations. They provide a more objective and less invasive experience for patients while also being more convenient for investigators. The captured images can be stored for future studies, and their magnification allows for enhanced detail analysis [18].

As people are afraid of exposure to infection after the COVID-19 pandemic, with their increased interest in using social media platforms and online educational methods, they prefer communicating with their doctors by sending photos and being diagnosed [19, 20]. Therefore, this study aims to assess the reliability of mobile photography in dental caries diagnosis in school children.

## Participants and methods

*Ethical approval and consent to participate.*

The Scientific Research Ethical Committee, Military Medical Academy, Egypt provided approval with number 12–2023 in compliance with the Helsinki Declaration. A written informed consent was obtained from parents/legal guardians regarding clinical examination and mobile photos of both arches of their children, as well as publication of photos without revealing their identity. The informed consent was sent to the parents through school administrators, and on the day of examination, all children who provided the written signed informed consent were included in the study. Collection of consents was performed by the examiner (E3). A verbal assent was obtained from children above 7 years of age for their participation in the study.

### Study design and settings

This diagnostic accuracy study was conducted in Lycée El Harm language private school, Giza, Egypt. This is an urban school. It has all grades from preschool to secondary. It includes two sections (the English and French sections). The total number of children in the primary (1–6) grades was 720 children. Children were invited from primary one to primary 6 to participate in both sections. The age of children in primary one was 6 to 7 years, primary two was 7 to 8 years, primary three was 8 to 9 years, primary four was 9 to 10 years, primary five was 10 to 11 years and primary six was 11 to 12 years. The study took place from April 2022 until December 2023.

### Participants

#### Inclusion criteria

Children between 6–12 years old with mixed dentition only and medically free from any systemic conditions were included.

#### Exclusion criteria

Uncooperative children and children with orthodontic appliances were excluded.

### Outcome

This study aimed to assess the accuracy and reliability of teledentistry mobile photos in detecting and diagnosing dental caries among school children in comparison to the reference standard (clinical examination).

**Sample size and sampling process:**

- *Sampling type*: convenience sample.
- *Sample size calculation:* Sample size calculation was performed using G*Power version 3.1.9.2, [21] University Kiel, Germany. Copyright (c) 1992–2014. The effect size d was 0.28 according to Subbalekshmi et al. [22], using alpha (α) level of 0.05 and Beta (β) level of 0.05, i.e., power = 95%; the estimated sample size (n) should be 140 students for this study.

### Methods

A total of 167 children joined the study; sixteen of them were excluded as they were above 12 years. The clinical photographs of ten children were used as a guide for intra-examiner and inter-examiner calibration. The study population (141 children) was divided into two major groups according to the school sections; the English and the French section, and each group was subdivided as per grade at school, from primary 1 to primary 6.

The first examiner (E1) clinically examined the children and evaluated the photos after 2 weeks wash-out period according to [13], the second examiner (E2) evaluated the photos only and did not examine the children while the third examiner (E3) captured the clinical photos without participation in the clinical examination process. Dental diagnostic charts were prepared to standardize data collection such as age, birthdate, gender, year of study, previous medical and dental history, and to record the caries experience using the WHO caries indices deft/DMFT (d = decayed tooth, e = decayed tooth indicated for extraction and f = filled tooth/ D = decayed tooth, M = missed tooth due to caries and F = filled tooth) [23]. Clinical dental examination was conducted in the classroom using daylight on the student chair by E1. This is the same condition for taking photographs by E3 using a mobile phone. The occlusal surfaces were examined for the presence of dental caries and no radiographs were taken.

### Calibration of examiners

#### Intra-examiner reproducibility

Ten excluded children were used as calibration for the examiner and to test and adjust the dimensions and quality of the photos by recording their caries indices clinically and through photos. E1 and E2 examined the children's photos at two different times. More than 85% matching results at baseline and after 48 h was the minimally accepted percentage for calibration.

#### Inter-examiner calibration

Similarly, as intra-examiner reproducibility, calibration was performed on both examiners.

This study was conducted in two phases. Phase I—gold standard phase: involved the clinical examination of children by E1. Pre-prepared charts were fulfilled including the demographics of the children with the caries index calculation. Then, E3 captured two photographs of both arches, from occlusal view using an iPhone 13Pro 12MP back camera in daylight**.** The idea behind using a mobile phone and daylight is to evaluate if the normal photos received daily from parents or patients are reliable or not. No special photography equipment was used in this study.

The caries indices used were (deft) and (DMFT) index for mixed dentition stage. Children were asked about their lost anterior teeth if present to assess the cause of tooth loss either due to trauma or caries.

Phase II – photographs management phase: involved the transferal of mobile photos captured at school to a laptop with an LCD 15-inch screen and a resolution of 1920 × 1080. The same photos were examined by both examiners on the same computer on separate occasions.

Three values of caries indices scores were generated for every child as follows:

[def/DMF-1—as examined and scored by E1 in clinical examination phase; def/DMF-2—as examined and scored by E1 (mobile photos) and def/DMF-3 —as examined and scored by E2 (mobile photos)].

After screening, an educational program was performed to all the children on how to brush their teeth properly, using a dental model and any child who needs treatment intervention was referred to either the Pediatric Dentistry and Dental Public Health Department, Faculty of Dentistry, Cairo University or to a private clinic near the school with a special offer to any child referred from the school.

### Statistical analysis

Categorical data were presented as frequency and percentage values. Numerical data were presented as mean, standard deviation, median, and interquartile range values. They were analyzed for normality using the Shapiro–Wilk test and were found to be non-parametric except for age. They were analyzed for intergroup comparisons using Freidman's test, followed by the Nemenyi post hoc test. Reliability was analyzed using weighted kappa. Internal consistency was analyzed using Cronbach's alpha. Diagnostic accuracy was determined using ROC curve analysis. The significance level was set at *p* ≤ 0.05 within all tests. Statistical analysis was performed with R statistical analysis software version 4.1.3 for Windows.^1^

## Results

### I-Demographic data

The total number of school children examined was 141 with an age range of 8.96 (± 1.66). Seventy-nine male and Sixty-two females were participated as shown in Table 1.

**Table 1:** Demographic data

Parameter			Value
**Sex**	**Male**	**n**	79
		**%**	56.0%
	**Female**	**n**	62
		**%**	44.0%
**Age (years)**	**Mean ± SD**		8.96 ± 1.66
**Grade**	**First**	**n**	38
		**%**	27.0%
	**Second**	**n**	28
		**%**	19.9%
	**Third**	**n**	26
		**%**	18.4%
	**Fourth**	**n**	27
		**%**	19.1%
	**Fifth**	**n**	12
		**%**	8.5%
	**Sixth**	**n**	10
		**%**	7.1%
**Section**	**French**	**n**	31
		**%**	22.0%
	**English**	**n**	110
		**%**	78.0%

### II-Clinical examination

Summary statistics for clinical examination are presented in Table 2. Majority of the children had previous dental treatment 87 (61.7%) and their intraoral examination was normal 138 (97.9%).

**Table 2:** Summary statistics for dental diagnosis

**Parameter**			**Value**
**Dental history**	**No previous treatment**	**n**	54
		**%**	38.3%
	**Previous treatment**	**n**	87
		**%**	61.7%
**Intraoral examination**	**Normal**	**n**	138
		**%**	97.9%
	**Abnormal**	**n**	0
		**%**	0.0%
	**Others**	**n**	3
		**%**	2.1%
**Clinical examination (E1)**	**def**	**Mean ± SD**	2.75 ± 2.57
		**Median (IQR)**	2.00(0.00–5.00)
	**DMF**	**Mean ± SD**	0.40 ± 0.95
		**Median (IQR)**	0.00(0.00–0.00)
**Photos examination (E1)**	**def**	**Mean ± SD**	2.79 ± 2.48
		**Median (IQR)**	2.00(1.00–5.00)
	**DMF**	**Mean ± SD**	0.44 ± 0.92
		**Median (IQR)**	0.00(0.00–0.00)
**Photos examination (E2)**	**def**	**Mean ± SD**	2.95 ± 2.65
		**Median (IQR)**	2.00(1.00–5.00)
	**DMF**	**Mean ± SD**	0.45 ± 0.91
		**Median (IQR)**	0.00(0.00–1.00)

### III-Diagnostic Accuracy for both examiners

#### Examiner 1

**Fig. 1:**
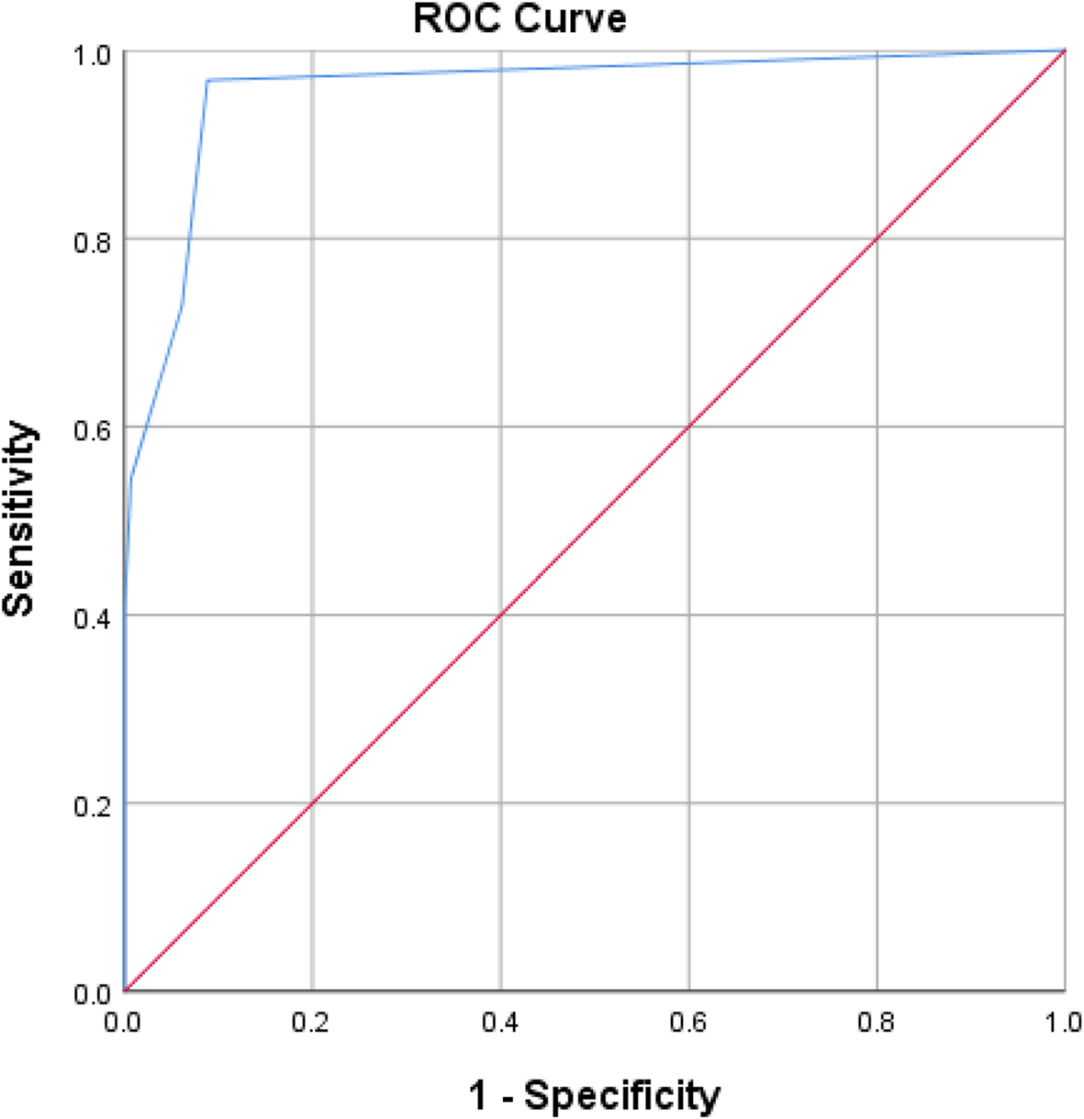
ROC curve for E1

Diagnostic accuracy for E1 is presented in Table 3 and the ROC curve in Fig. 1. ROC curve analysis revealed that sensitivity was (96.8%), specificity was (91.2%), positive predictive value was (90.3%), negative predictive value was (97.1%) and the area under the ROC curve (AUC) was 0.958 with 95% Confidence Interval of (0.934– 0.982).

**Table 3:** Diagnostic accuracy for E1

Sensitivity	Specificity	Positive predictive value	Negative predictive value	AUC	95% CI
96.8%	91.2%	90.3%	97.1%	0.958	0.934 to 0.982

#### Examiner 2

**Fig. 2:**
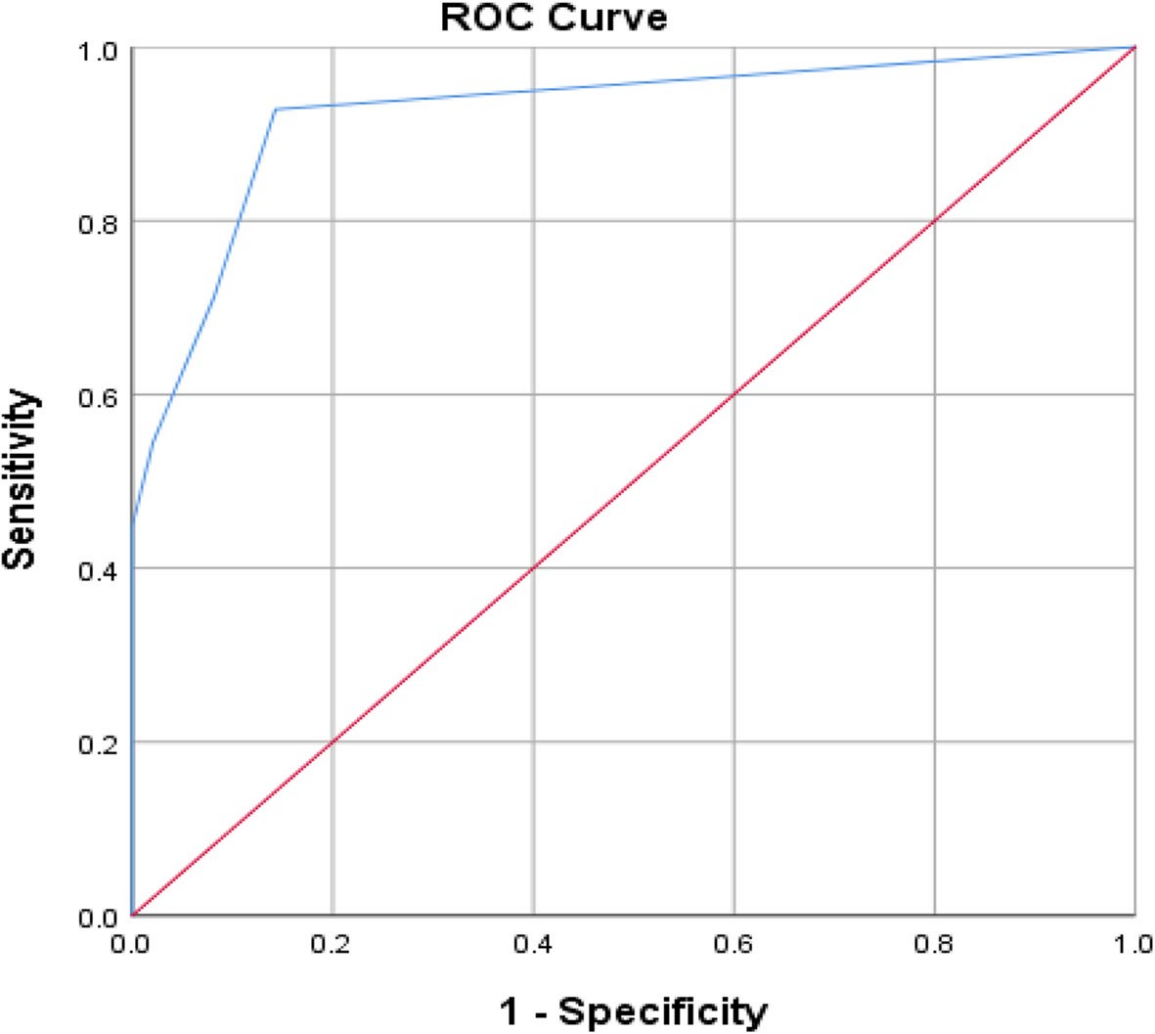
ROC curve for E2

Diagnostic accuracy for E2 is presented in Table 4 and the ROC curve in Fig. 2. ROC curve analysis revealed that sensitivity was (92.8%), specificity was (85.7%), positive predictive value was (84.7%), negative predictive value was (93.3%) and the area under the ROC curve (AUC) was 0.925 with 95% Confidence Interval of (0.892– 0.958).

**Table 4:** Diagnostic accuracy for E2

Sensitivity	Specificity	Positive predictive value	Negative predictive value	AUC	95% CI
92.8%	85.7%	84.7%	93.3%	0.925	0.892 to 0.958

#### IV-Reliability of both examiners

Reliability analysis is presented in Table 5**.** There was a strong agreement between clinical and photos (E1) (kw = 0.899, *p* < 0.001), clinical and photos (E2) (kw = 0.834, *p* < 0.001), and inter-observer (kw = 0.898, *p* < 0.001).

**Table 5:** Reliability analysis

Parameter		Agreement	No agreement	Weighted Kappa	*p*-value
**Clinical versus photos (E1)**	**n**	112	29	**0.899**	< 0.001*
	**%**	79.4%	20.6%		
**Clinical versus photos (E2)**	**n**	93	48	**0.834**	< 0.001*
	**%**	66.0%	34.0%		
**Inter-observer**	**n**	108	33	**0.898**	< 0.001*
	**%**	76.6%	23.4%		

^*^Significant (*p* ≤ 0.05) ns; non-significant (*p* > 0.05)

#### V-Internal consistency

Internal consistency analysis is presented in Table 6. For DMF score, the internal consistency between clinical and photos (E2) was acceptable 0.790(0.706:0.85). For other scores, the internal consistency between all variables was excellent (α > 0.9).

**Table 6:** Internal consistency analysis

Measurement	Method	Mean ± SD	Cronbach's alpha (95%CI)
**def**	**Clinical examination**	2.77 ± 2.56	**0.983(0.976:0.988)**
	**Photos (E1)**	2.79 ± 2.48	
	**Clinical examination**	2.77 ± 2.56	**0.966(0.953:0.976)**
	**Photos (E2)**	2.99 ± 2.65	
	**Photos (E1)**	2.79 ± 2.48	**0.977(0.968:0.984)**
	**Photos (E2)**	2.99 ± 2.65	
	**Clinical examination**	2.77 ± 2.56	**0.983(0.978:0.988)**
	**Photos (E1)**	2.79 ± 2.48	
	**Photos (E2)**	2.99 ± 2.65	
**DMF**	**Clinical examination**	0.40 ± 0.95	**0.917(0.884:0.94)**
	**Photos (E1)**	0.44 ± 0.92	
	**Clinical examination**	0.40 ± 0.95	**0.790(0.706:0.85)**
	**Photos (E2)**	0.45 ± 0.91	
	**Photos (E1)**	0.44 ± 0.92	**0.915(0.882:0.94)**
	**Photos (E2)**	0.45 ± 0.91	
	**Clinical examination**	0.40 ± 0.95	**0.914(0.886:0.936)**
	**Photos (E1)**	0.44 ± 0.92	
	**Photos (E2)**	0.45 ± 0.91	

#### Case examples

**Fig. 3:**
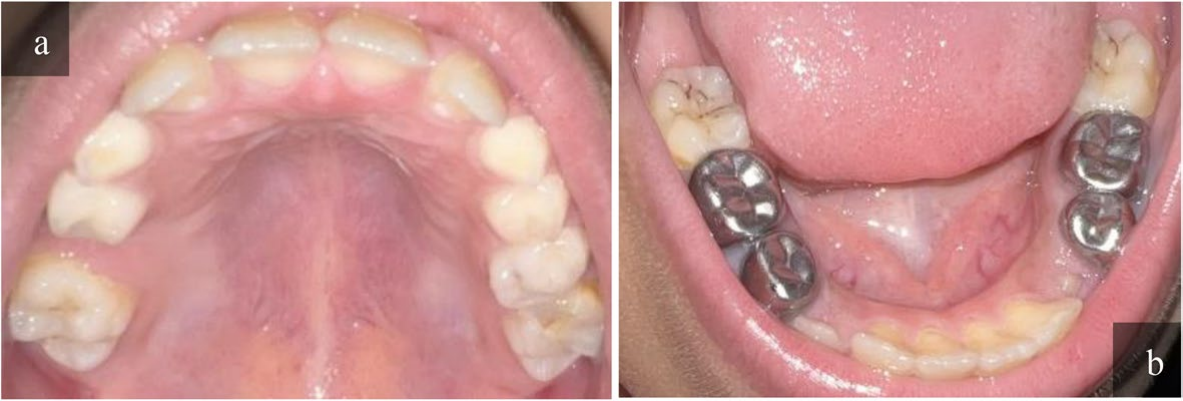
Clinical photographs for both arches showing an 8-years old girl, in Grade 1. She had previous dental visits. Intra and extra oral examinations were performed, soft tissue examination was within normal, her clinical examination showed a deft = 5 and DMFT = 0. In teledental examination, E1 and E2 agreed on her deft = 5, but there was a conflict in her DMFT due to the presence of stains on her lower 6s. E1 scored a DMFT = 1 and E2 scored a DMFT = 2

**Fig. 4:**
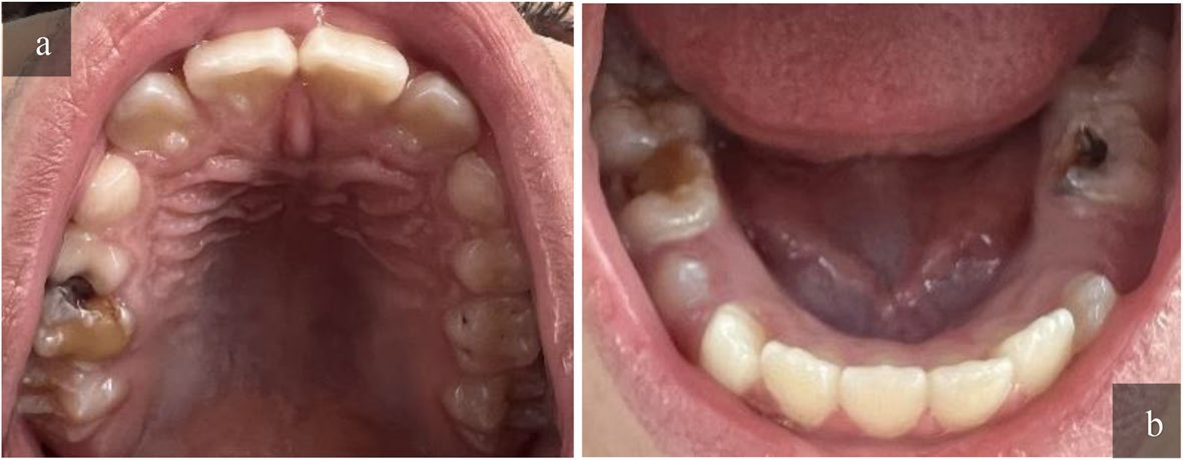
Clinical photographs for both arches showing a 10-years-old boy, in Grade 4. He had previous dental experience. Intra and extra oral examination were performed, soft tissue examination was within normal, his clinical examination showed a deft = 4, DMFT = 0 and this agreed with both examiners

Two cases are presented in Figs. 3 and 4 as an example of intraoral photographs captured at school with a mobile phone.

## Discussion

The strengths of this study include being a diagnostic accuracy study with all parameters calculated. The use of a standardized mobile and computer for assessing photos. The presence of strict criteria in intra-examiner reproducibility and inter-examiner calibration. Lastly, the study was conducted on a large group of school children.

The limitations were, firstly, no radiographs were taken for children, which could underdiagnose and miss interproximal caries. Secondly, study design excludes uncooperative and children with medical conditions that may have a higher caries risk than study population. Thirdly, the presence of stains that might be misdiagnosed with dental caries especially in first permanent molar. Finally, we did not use any imaging retractors to simulate the photos provided by parents. All photos were provided under daylight with no lighting accessories.

Our study aimed to assess caries detection accuracy and reliability by examining intraoral photographs compared with clinical examinations. All occlusal surface photos were only included in the analysis and there was no exclusion to any occlusal surfaces.

Our results showed that teledentistry has reliable accuracy in dental caries detection. There was no statistically significant difference between the mean deft/DMFT of the photo’s assessment and the clinical examination.

The sensitivity of photos assessment describes the accuracy to identify participants with caries. While the specificity is how correct it is to identify participants free from dental caries.

Therefore, it’s important to connect them with their positive and negative predictive values (PPV and NPV) [15], where PPV avoids reporting false-positive cases and NPV avoids reporting false-negative values.

In our study, all the PPVs and NPVs for both examiners ranged from 84.7 to 97.1. This explains the previously mentioned interpretation of the accuracy of the sensitivity and specificity scores [15, 18, 24]

Regarding sensitivity and specificity, photos assessment showed acceptable sensitivity and specificity when compared to clinical examination. The current study showed that there was a higher sensitivity for both examiners 96.8% and 92.8% than specificity 91.2% and 85.7% for E1 and E2, respectively, which is similar to findings by [15, 24, 25].

The quality of photos obtained in our study might be due to the fact of using an advanced smartphone with enhanced specifications. Also, the standardization of interpretation of photos on the same computer helped in improving the final presentation of the photos. This is in accordance to AlShaya et al. [15].

Our results showed greater sensitivity than specificity, this is similar to Kopycka-Kedzierawski et al. [13], and AlShaya et al. [15]. False negative results in our study was higher than false positive in disagreement with AlShaya et al. [26].

In our study, reliability of photos assessment is better in primary teeth than in permanent teeth due to the presence of stains in the permanent molars which might be seen as dental caries in the photos. It also showed high PPV and NPV for clinical examination only, indicating the ability to avoid false results similar to [15]. In our study, the sensitivity was higher than specificity in contrast with Morosini et al. and Estai et al. [25, 27], where specificity was higher than sensitivity.

No statistically significant differences exist between the photos scores and the clinical examinations. The photos are like visual diagnosis with less examiner’s bias, digital scoring, and archiving. This was in accordance with Boye et al. [24].

## Conclusions

We conclude that teledentistry screening photograph examinations can provide accurate and reliable results as conventional visual clinical examinations regarding occlusal caries detection and diagnosis without dental radiographs. With improved camera specifications, higher quality images offer more precise results. Therefore, teledentistry is a valuable tool for school-screening programs improving the overall oral health of school children. Large-scale screenings can be easily conducted by being more practical and cost efficient.

## Data Availability

Data will be available upon request from the corresponding author.

## Footnotes

1 R Core Team (2022). R: A language and environment for statistical computing. R Foundation for Statistical Computing, Vienna, Austria. URL https://www.R-project.org/.

## References

[1] Chin JR, Kowolik JE, Stookey GK. Dental caries in the child and adolescent. In McDonald and Avery’s Dentistry for the Child and Adolescent, 10th ed. Elsevier; 2016. p. 155–76.

[2] Pitts NB, Zero DT, Marsh PD, Ekstrand K, Weintraub JA, Ramos-Gomez F, et al. Dental caries. Nat Rev Dis Primers. 2017;3:17030:1–16. 10.1038/nrdp.2017.30 28540937

[3] Banakar S, Keshavarz K. An Investigation on Relationship between Prevalence of Dental Caries and Underweight in 6–10 Year Old Children in Gachsaran. J Dent. 2005;6:10–6.

[4] Sheiham A. Dental caries affects body weight, growth and quality of life in pre-school children. Br Dent J. 2006;201:625–6. 17128231 10.1038/sj.bdj.4814259

[5] Ramos-Jorge J, Pordeus IA, Ramos-Jorge ML, Marques LS, Paiva SM. Impact of untreated dental caries on quality of life of preschool children: different stages and activity. Community Dent Oral Epidemiol. 2014;42:311–22. 24266653 10.1111/cdoe.12086

[6] Wang Y, Zhang Y, Pan T, Lin H, Zhou Y. Metabolic differences of the oral microbiome related to dental caries - A pilot study. Arch Oral Biol. 2022;141: 105471. 35689993 10.1016/j.archoralbio.2022.105471

[7] Singh N, Sultan A, Juneja A, Aggarwal I, Palkit T, Ohri T. Integration of teledentistry in oral health care during COVID-19 pandemic. Saint’s Int Dent J. 2020;4:77.

[8] Da Costa CB, Peralta FDS, Ferreira De Mello ALS. How Has Teledentistry Been Applied in Public Dental Health Services? An Integrative Review. 2020;26:945–54. https://home.liebertpub.com/tmj. 10.1089/tmj.2019.0122 31573410

[9] Xiao J, Luo J, Ly-Mapes O, Wu TT, Dye T, Al Jallad N, et al. Assessing a Smartphone App (AICaries) That Uses Artificial Intelligence to Detect Dental Caries in Children and Provides Interactive Oral Health Education: Protocol for a Design and Usability Testing Study. JMIR Res Protoc. 2021;10: e32921. 34529582 10.2196/32921 PMC8571694

[10] Cooper BR, Engeswick LM. Knowledge, attitudes, and confidence levels of dental hygiene Students regarding teledentistry. Am Dent Hyg Assoc. 2007;81:114.

[11] Kopycka-Kedzierawski DT, Billings RJ. Prevalence of dental caries and dental care utilisation in preschool urban children enrolled in a comparative-effectiveness study. Eur Arch Paediatr Dent. 2011;12:133–8. 21640057 10.1007/BF03262794 PMC3111947

[12] Kopycka-Kedzierawski DT, Billings RJ. Teledentistry in inner-city child-care centres. 2016;12:176–81. 10.1258/135763306777488744. 10.1258/135763306777488744 16774697

[13] Kopycka-Kedzierawski DT, Billings RJ, McConnochie KM. Dental screening of preschool children using teledentistry: a feasibility study. Pediatr Dent. 2007;29:209–13. 17688017

[14] Panat SR, Chakarvaty A, Aggarwal A. Teledentistry: A new revolution. J Dent Sci Oral Rehabil. 2012;3:1–4.

[15] AlShaya M, Farsi D, Farsi N, Farsi N. The accuracy of teledentistry in caries detection in children - A diagnostic study. Digit Heal. 2022;8:1–14. 10.1177/20552076221109075 PMC9237921 35774249

[16] Almalki M, FitzGerald G, Clark M. Health care system in Saudi Arabia: an overview. EMHJ-Eastern Mediterr Heal Journal. 2011;17(10):784–93. 10.26719/2011.17.10.784 22256414

[17] Summerfelt FF. Teledentistry-Assisted, Affiliated Practice for Dental Hygienists: An Innovative Oral Health Workforce Model. J Dent Educ. 2011;75:733–42. 21642518

[18] Elfrink MEC, Veerkamp JSJ, Aartman IHA, Moll HA, Ten Cate JM. Validity of scoring caries and primary molar hypomineralization (DMH) on intraoral photographs. Eur Arch Paediatr Dent. 2009;10:5–10. 19863892 10.1007/BF03262693

[19] Mahdavi A, Atlasi R, Naemi R. Teledentistry during COVID-19 pandemic: scientometric and content analysis approach. BMC Health Serv Res. 2022;22:1111. 36050678 10.1186/s12913-022-08488-z PMC9436727

[20] Deshpande S, Patil D, Dhokar A, Bhanushali P, Katge F. Teledentistry: A boon amidst COVID-19 lockdown—A narrative review. Int J Telemed Appl. 2021;2021:8859746. 33628231 10.1155/2021/8859746 PMC7894051

[21] Faul F, Erdfelder E, Lang A-G, Buchner A. G* Power 3: A flexible statistical power analysis program for the social, behavioral, and biomedical sciences. Behav Res Methods. 2007;39:175–91. 17695343 10.3758/bf03193146

[22] Subbalekshmi T, Anandan V, Apathsakayan R. Use of a Teledentistry-based Program for Screening of Early Childhood Caries in a School Setting. Cureus. 2017;9:1–7. Available from: https://pubmed.ncbi.nlm.nih.gov/28875089/. Cited 2024 Oct 28. 10.7759/cureus.1416 PMC5580976 28875089

[23] Petersen PE, Bourgeois D, Ogawa H, Estupinan-Day S, Ndiaye C. The global burden of oral diseases and risks to oral health. Bull World Health Organ. 2005;83:661–9. 16211157 PMC2626328

[24] Boye U, Willasey A, Walsh T, Tickle M, Pretty IA. Comparison of an intra-oral photographic caries assessment with an established visual caries assessment method for use in dental epidemiological studies of children. Community Dent Oral Epidemiol. 2013;41:526–33. 23566100 10.1111/cdoe.12049

[25] Estai M, Kanagasingam Y, Mehdizadeh M, Vignarajan J, Norman R, Huang B, et al. Mobile photographic screening for dental caries in children: Diagnostic performance compared to unaided visual dental examination. J Public Health Dent. 2022;82:166–75. 33495989 10.1111/jphd.12443

[26] AlShaya MS, Assery MK, Pani SC. Reliability of mobile phone teledentistry in dental diagnosis and treatment planning in mixed dentition. J Telemed Telecare. 2020;26:45–52. 30134778 10.1177/1357633X18793767

[27] Morosini IDAC, De Oliveira DC, Ferreira FDM, Fraiz FC, Torres-Pereira CC. Performance of distant diagnosis of dental caries by teledentistry in juvenile offenders. Telemed e-Health. 2014;20:584–9. 10.1089/tmj.2013.0202 24693859

